# Interleukin-10 protects against aging-induced endothelial dysfunction

**DOI:** 10.1002/phy2.149

**Published:** 2013-11-11

**Authors:** Dale A Kinzenbaw, Yi Chu, Ricardo A Peña Silva, Sean P Didion, Frank M Faraci

**Affiliations:** 1 Department of Internal Medicine, Cardiovascular Center, Carver College of Medicine, University of IowaIowa City, 52242, Iowa; 2 Department of Pharmacology, University of Mississippi Medical CenterJackson, 39216, Mississippi; 3 Department of Pharmacology, Cardiovascular Center, Carver College of Medicine, University of IowaIowa City, 52242, Iowa

**Keywords:** Endothelium, interleukin-6, NADPH oxidase, nitric oxide, oxidative stress

## Abstract

Carotid and cerebrovascular disease increase markedly with age contributing to stroke and cognitive impairment. Inflammation is a key element of vascular disease. In these studies, we tested the hypothesis that interleukin-10 (IL-10), a potent anti-inflammatory cytokine, protects against aging-induced endothelial dysfunction. Responses of carotid arteries from adult (5 ± 1 months) and old (22 ± 1 months) wild-type and IL-10-deficient mice were examined in vitro. Acetylcholine (an endothelium-dependent agonist) produced relaxation in arteries from adult wild-type that was not altered in old mice. In contrast, relaxation to acetylcholine in arteries from old IL-10-deficient mice was reduced by ∼50% (*P* < 0.05). Tempol, a scavenger of superoxide, did not affect responses in adult or old wild-type mice, but restored vasodilation to acetylcholine to normal in old IL-10-deficient mice. Responses of the carotid artery to nitroprusside (an endothelium-independent agonist) were not altered in any group. Vascular expression of IL-6 (a proinflammatory mediator of vascular disease) and components of NADPH oxidase (a major source of superoxide) was increased in old IL-10-deficient mice compared with wild-type (*P* < 0.05). These findings provide the first evidence that age-related and superoxide-mediated endothelial dysfunction occurs earlier with IL-10 deficiency. Our findings suggest a novel role for IL-10 to protect against age-related increases in expression of IL-6, oxidative stress, and endothelial dysfunction.

## Introduction

Aging is the single greatest risk factor for vascular disease (Lakatta and Levy 2003). Starting at approximately the sixth decade of life in humans, the rate of vascular events including stroke increases markedly with age (Rothwell et al. 2005). Carotid artery and cerebrovascular disease greatly increase the risk for ischemic stroke and contribute to cognitive impairment (Lorenz et al. 2007; Wendell et al. 2009; Arntzen and Mathiesen 2011). Despite this impact, relatively little is known regarding the vascular biology of aging as the vast majority of experimental studies have used young or adult models when studying either blood vessels or cells in culture.

Endothelial dysfunction is a key event for both the onset and the progression of vascular disease (Faraci 2011b; Libby et al. 2011). A major component of this dysfunction is the loss of nitric oxide (NO)-mediated signaling that originates normally in endothelial cells and exerts diverse protective effects within the vessel wall and on nearby target cells (Faraci 2011b; Fogel and Pober 2012). Endothelium-dependent and NO-mediated vasodilation decreases with age in both experimental models and in humans (Brown et al. 2006, 2007; Park et al. 2007; Mayhan et al. 2008; Modrick et al. 2009; Rodriguez-Manas et al. 2009; Faraci 2011a,b; El Assar et al. 2012).

Inflammation plays a major role in vascular disease (Libby et al. 2011; Tabas and Glass 2013). Activation of inflammatory-related signaling occurs in vascular cells in humans with cardiovascular disease as well as experimental models used to study the impact of cardiovascular risk factors (Donato et al. 2007; Rodriguez-Manas et al. 2009; El Assar et al. 2012; Tabas and Glass 2013). Inflammatory-dependent mechanisms are propagated via intermediate molecules including reactive oxygen species and proinflammatory cytokines (Didion et al. 2009; Johnson et al. 2013; Tabas and Glass 2013). For example, recent studies highlight the importance of interleukin-6 (IL-6) and IL-6 dependent signaling in vascular disease (Rodriguez-Manas et al. 2009; Boekholdt and Stroes 2012; Miwa et al. 2013). While some molecules promote immune-related responses, the anti-inflammatory cytokine IL-10 decreases the magnitude of proinflammatory responses and promotes resolution of inflammation (Ouyang et al. 2011; Tabas and Glass 2013).

Despite evidence that elements of immune-related signaling play a key role in vascular disease, our understanding of mechanisms that regulate these processes is limited, particularly during aging. In this study, we examined the hypothesis that IL-10 normally protects against endothelial dysfunction during aging. To test the hypothesis, we used a mouse model of aging and focused on changes in endothelial function. As noted above, endothelial dysfunction is a fundamental element of vascular disease. Atherosclerosis is an advanced form of vascular disease and endothelial dysfunction is a major contributor to this process. We study carotid arteries because this segment of the vasculature is a common site of development of atherosclerosis and is a site where the clinical consequences of vascular disease are most apparent (Libby et al. 2011). Thus, we are studying mechanisms that underlie early changes in a model of vascular disease. Our findings indicate that genetic deficiency in IL-10 enhanced expression of IL-6 and accelerated endothelial dysfunction suggesting IL-10 normally protects against age-induced endothelial dysfunction.

## Methods

### Animals

IL-10-deficient mice (*IL-10*^*−/−*^) used in these studies have been backcrossed more than 12 generations onto the C57BL/6 strain and thus C57BL/6 mice were used as wild-type controls. Mice were fed regular chow and water was available ad libitum. All experimental protocols were in accordance with the *Guide for the Care and Use of Laboratory Animals* (National Institutes of Health) and approved by the Institutional Animal Care and Use Committee at the University of Iowa.

Because we observed no apparent sex-related differences in these experiments, results from both male and female mice were combined. Mice were studied at 5 ± 1 (adult) or 22 ± 1 months of age (old). Body weight was similar in adult wild-type and IL-10-deficient mice: 30.4 ± 1.2 and 27.9 ± 1.5 g, respectively. With aging, body weight was maintained in wild-type mice (31.2 ± 1.4 g), but was decreased somewhat in old IL-10-deficient mice (20.1 ± 0.6 g, *P* < 0.05).

### Measurements of vascular responses

Mice were killed with pentobarbital (∼100 mg/kg, i.p.). Vessels were removed, cleaned of loose connective tissue, cut into rings and placed into individual organ baths for measurement of isometric tension (contraction and relaxation). To evaluate endothelial function (Faraci et al. 1998; Lamping and Faraci 2001), responses to acetylcholine were measured in carotid arteries following submaximal precontraction (∼50–60% of maximum) using U46619 (9,11-dideoxy-11a,9a-epoxy-methanoprostaglandin F_2*α*_). In these arteries, vasodilation to acetylcholine is mediated by endothelium-derived NO activating soluble guanylate cyclase (Faraci et al. 1998). Nitroprusside is a NO donor and was used to assess endothelium-independent relaxation. Tempol (1 mmol/L), a superoxide scavenger, was used to determine if vascular responses were affected by superoxide. A full dose–response curve to U46619 was obtained at the end of each protocol. We used U46619 because it produces very stable preconstriction in arteries from mice. In addition, this agent is an analogue of thromboxane A_2_, an important mediator of vascular effects of aggregating platelets.

### Quantitative real-time RT-PCR

RNA from aorta was prepared using the RNAeasy (Qiagen, Germantown, MD) method following extraction with TRIzol reagent (Invitrogen, Carlsbad, CA) (Chu et al. 2002). RNA concentrations were determined using a NanoDrop spectrophotometer, with an OD260/OD280 ratio of greater than 1.9 (indicating very high-quality RNA). Purified RNA (300 ng) was used for reverse transcription reaction (RT) (Chu et al. 2002; Modrick et al. 2012). Identical amounts of RT product were used for real-time PCR with a single well of a 96-well plate containing both TaqMan probes/primers (Applied Biosystems, Foster City, CA) for genes of interest [with carboxyfluorescein (FAM) fluorophor] and using β-actin (with VIC fluorophor) as a house-keeping gene. Expression levels were normalized to β-actin (4352341E). Relative expression levels were obtained using the ΔΔCt method as described (Chu et al. 2002). Expression of IL-6 (TaqMan primers/probe # Mm00446190_m1), tumor necrosis factor-*α* (TNF*α*, Mm004 43258_m1), suppressor of cytokine signaling-3 (SOCS3, Mm.PT.51.7804681), signal transducer and activator of transcription 3 (STAT3, Mm.PT.51.16704475), subunits of NADPH oxidases [Nox2 (Mm00432775_m1), and p22^phox^ (Mm00514478_m1), a major source of reactive oxygen species], as well as endothelial NO synthase (eNOS, Mm00435204_m1) were determined by quantitative real-time RT-PCR using the TaqMan method (Chu et al. 2002). Because angiotensin II contributes to vascular dysfunction with aging (Modrick et al. 2009) and can promote inflammation (Didion et al. 2009), we also measured expression of receptors for angiotensin II [AT_1_ (Mm01166161_m1) and AT_2_ (Mm01341373_m1)].

### Drugs

Acetylcholine, nitroprusside, and tempol were obtained from Sigma (St. Louis, MO) and were dissolved in saline. U46619 (Cayman Chemical, Ann Arbor, MI) was dissolved in ethanol with subsequent dilutions made in saline.

### Statistics

All values are ± SEM. Statistical analysis was performed using repeated measures analysis of variance (ANOVA) followed by the Tukey or Student–Newman–Keuls post hoc test to detect individual differences. A *P* < 0.05 was considered significant.

## Results

**Figure 1:**
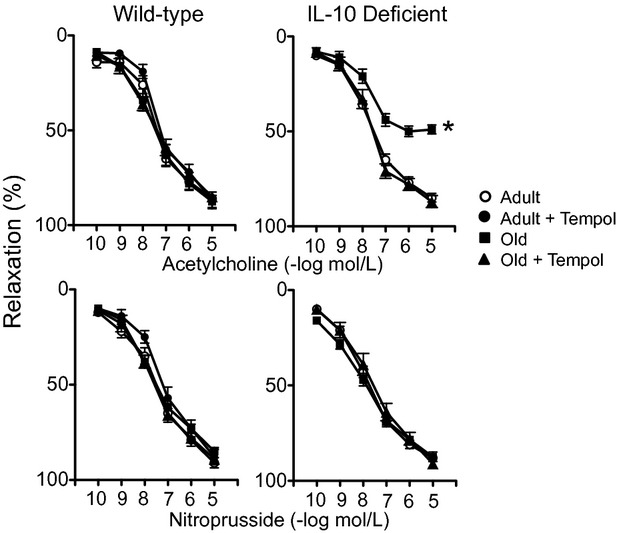
Responses of carotid arteries to acetylcholine (upper panels) and nitroprusside (lower panels) and effects of tempol in adult and old wild-type and IL-10-deficient mice. Values are means ± SE. *P < 0.05 versus wild-type. *N* = 7–10 in each group.

The endothelium-dependent agonist acetylcholine produced concentration-dependent relaxation of carotid arteries. Compared with wild-type adults, vascular responses to acetylcholine were not significantly altered in old wild-type mice (Fig. 1). Relaxation of the carotid artery to acetylcholine was similar in adult wild-type and adult IL-10-deficient mice. In contrast, responses to acetylcholine were reduced by ∼50% in old IL-10-deficient mice (Fig. 1). Relaxation of carotid arteries to nitroprusside was similar in all groups and was not affected by age or genotype (Fig. 1). The latter findings suggest that the dysfunction observed occurred at the level of endothelium and not vascular muscle. Thus, there was no evidence for endothelial dysfunction in carotid arteries from old wild-type mice. In contrast, there was substantial impairment of endothelial function with age in old IL-10-deficient mice.

In wild-type mice, contraction of the carotid artery to the thromboxane agonist U46619 was not affected by age. Responses to U46619 tended to increase in old IL-10-deficient mice, but these differences were not statistically significant (data not shown).

Tempol did not alter responses to acetylcholine in adult or old wild-type mice (Fig. 1). In contrast, relaxation of carotid arteries to acetylcholine in old IL-10-deficient mice was increased by tempol to levels seen in adult and old wild-type (Fig. 1). Regardless of age or genotype, vasodilation to nitroprusside was not affected by tempol (Fig. 1). Similarly, tempol did not affect vasoconstrictor responses to U46619 in old wild-type or old IL-10-deficient mice (data not shown).

**Figure 2:**
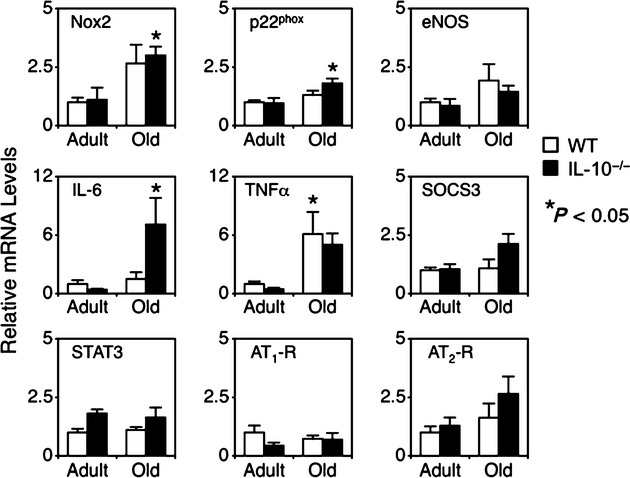
Vascular expression of mRNA from adult and old wild-type (WT) and IL-10-deficient (IL-10^−/−^) mice. Expression of TNF*α* increased significantly in old WT mice, but was not changed any further in old mice deficient in IL-10. Expression of Nox2, p22^phox^, and IL-6 was increased significantly in old IL-10-deficient mice compared with adult WT (*P < 0.05 vs. adult WT). Compared with adult wild-type mice, there were no significant changes in expression of eNOS, STAT3, AT_1_, or AT_2_ receptors in old IL-10-deficient mice. *N* = 7–9 in each group.

To gain additional insight into mechanisms that may contribute to vascular aging and endothelial dysfunction, we measured expression of several genes previously implicated in vascular inflammation and oxidative stress (Fig. 2). There were no significant differences in expression of these genes in adult wild-type versus adult IL-10-deficient mice (Fig. 2). Compared with adult wild-type mice, levels of mRNA for TNF*α* increased in old wild-type mice compared with adults, but did not change any further in IL-10-deficient animals. Thus, aging alone was sufficient to increase TNF*α*, and this effect was not modulated by the absence of IL-10. Vascular expression of Nox2 tended to increase with aging in wild-type mice, but this change was not statistically significant. In IL-10-deficient mice, increases in Nox2 with aging were significant. Changes in p22^phox^ (another membrane component of NADPH oxidase) were not detected in old wild-type, but were increased in old IL-10-deficient animals. Expression of IL-6, a cytokine implicated in vascular disease and hypertension, was not altered in old wild-type, but was increased substantially in old IL-10 knockout mice. Lastly, compared with adult wild-type mice, there were no significant changes in expression of eNOS, STAT3, AT_1_, or AT_2_ receptors in old IL-10-deficient mice.

## Discussion

There are several new findings in this study. Genetic deficiency in IL-10 did not alter vascular function in adult mice, but greatly increased endothelial dysfunction in carotid arteries in old mice. Impairment of endothelium-dependent relaxation in old mice lacking IL-10 was reversed by a scavenger of superoxide suggesting a key role for oxidative stress. Changes in vascular function in old IL-10-deficient mice were not associated with changes in expression of eNOS, but were accompanied by increased expression of IL-6 and components of NADPH oxidase – known mediators of vascular disease. Overall, our findings provide direct evidence that IL-10 plays a major role to suppress age-induced oxidative stress, increases in IL-6 expression, and endothelial dysfunction.

We focused on IL-10 and its potential impact in a model of aging for several reasons. While proinflammatory mechanisms are known to promote vascular disease (Libby et al. 2011; Tabas and Glass 2013), the importance of endogenous mechanisms that may limit vascular inflammation are much less clear. Previous research suggested IL-10 may play a key role in this regard. In nonvascular cells, a major role for IL-10 is to inhibit expression of proinflammatory cytokines including IL-6 (Ouyang et al. 2011). While the impact of IL-10 in both the innate and the adaptive immune system are known, the impact of IL-10 for vascular biology has only begun to emerge. Nuclear factor (NF)-*κ*B (NF-*κ*B) is a transcription factor involved in the regulation of many inflammatory-related genes including IL-6 (Brasier 2010). Activation of NF-*κ*B along with components of inflammatory signaling pathways occurs in vascular cells with aging (Donato et al. 2007; El Assar et al. 2012). Through effects on inhibitor of *κ*B (I*κ*B) activity, NF-*κ*B degradation, and DNA-binding activity, IL-10 is a potent inhibitor of NF-*κ*B-mediated effects (Ouyang et al. 2011).

Vascular function and related endpoints appear to be normal in adult IL-10-deficient mice under control conditions. In combination with previous work (Gunnett et al. 1999, 2000, 2002; Didion et al. 2009), the present findings suggest that expression of eNOS, levels of superoxide, vasodilator responses, and vasoconstrictor responses are similar in adult wild-type mice and adult mice lacking IL-10. We and others have found that blood pressure in wild-type mice does not change significantly with aging (Pena Silva et al. 2012; Toth et al. 2013). Similarly, we have shown previously that arterial pressure is similar in adult wild-type and IL-10-deficient mice (Didion et al. 2009). For these reasons, we did not measure blood pressure in the old IL-10-deficient mice. While we assume based on previous work that blood pressure did not change with age, we cannot exclude the possibility that increases in arterial pressure occurred in old IL-10-deficient mice. If arterial pressure increased in these mice, such changes could have contributed to the accelerated endothelial function seen with aging.

The presence and degree of age-associated endothelial impairment is dependent on the vessel studied and the age at which the studies are performed. In mouse aorta and carotid artery (the vessel used in these experiments), endothelial function is unchanged or impaired only modestly at 22–24 months of age (Didion et al. 2006; Brown et al. 2007; Modrick et al. 2012). The finding in this study that endothelial function in carotid arteries was relatively normal in ∼22-month-old wild-type mice is consistent with the literature. Endothelial dysfunction does occur in these blood vessels (including the carotid artery) if wild-type mice are studies at age beyond ∼24 months (from ∼25–32 months of age)(Brown et al. 2007; Lund et al. 2009; Fleenor et al. 2012; Pena Silva et al. 2012; Walker et al. 2013). Thus, based on our own experience and the literature, we expected little impairment of endothelial function in wild-type mice when studied at ∼22 months of age. On the basis of this expectation, we thought the experimental design used here was appropriate in that it allowed us to test the hypothesis that IL-10 deficiency would augment age-related vascular dysfunction. Alternately, we could have studied mice at >24 months of age. However, the presence of a large effect of age per se on endothelial function in wild-type mice would potentially make it more difficult to detect further dysfunction (and thus test our hypothesis). Based on these issues, the study of mice at ∼22 months of age seemed reasonable to us. Our findings in this study indicate that the presence of IL-10 deficiency results in the appearance of prominent endothelial dysfunction at an earlier age.

The major endothelium-derived relaxing factor in the carotid artery is NO (Faraci et al. 1998; Lamping and Faraci 2001). Responses of this vessel to acetylcholine, which are mediated by NO (Faraci et al. 1998; Lamping and Faraci 2001), were greatly impaired in old IL-10-deficient mice, but could be restored to normal by acute administration of a scavenger of superoxide anion. The chemical interaction between NO and superoxide (and resulting inactivation of NO) is a major cause of impaired NO-mediated signaling and endothelial dysfunction in a variety of experimental models and in humans with vascular disease (Faraci 2011b). For example, endothelium-dependent responses in small mesenteric arteries and resistance vessels of the forearm in humans are mediated by NO normally, but impaired with aging (Lauer et al. 2001; Rodriguez-Manas et al. 2009; Angulo et al. 2012; Wray et al. 2012). These reduced responses are improved by scavenging superoxide or treatment with other antioxidants (Rodriguez-Manas et al. 2009; Angulo et al. 2012; Wray et al. 2012). In another study of effects of aging, we provided evidence that scavenging superoxide restores endothelial function (responses to endothelium-dependent agonists) by protecting NO (Modrick et al. 2009). On the basis of this and other previous work (Brown et al. 2006, 2007; Didion et al. 2006; Lund et al. 2009; Modrick et al. 2009), we assume that elevated superoxide was present and was responsible for impairing NO-mediated vasodilation in old IL-10-deficient mice. Thus, the finding in this study that superoxide is a key player in vascular aging in old IL-10-deficient mice is consistent with data in general that oxidative stress may be an important component of vascular aging. Despite the chronic nature of oxidative stress during aging, it is interesting that acute treatment with antioxidants is sufficient to restore endothelial function in both animal models of aging as well as elderly people (Didion et al. 2006; Mayhan et al. 2008; Modrick et al. 2009; Wray et al. 2012). Such results suggest that the vascular abnormalities observed are due to ongoing mechanisms rather than permanent changes in vascular cells.

A prominent source of superoxide in vascular cells is NADPH oxidase (Drummond et al. 2011). The importance of oxidative stress and NADPH oxidase in vascular abnormalities in models of aging as well as in vessels from older humans has been emphasized (Brown et al. 2006, 2007; Park et al. 2007; Mayhan et al. 2008; Rodriguez-Manas et al. 2009; Fleenor et al. 2012). Levels of superoxide and expression of components of NADPH oxidase in the vasculature increase with aging (Brown et al. 2006; Didion et al. 2006; Park et al. 2007; Mayhan et al. 2008; Rodriguez-Manas et al. 2009; Fleenor et al. 2012). Vascular dysfunction with aging is prevented by scavenging superoxide or genetic deletion of the Nox2 component of NADPH oxidase (Brown et al. 2006; Didion et al. 2006; Park et al. 2007; Mayhan et al. 2008; Modrick et al. 2009; Rodriguez-Manas et al. 2009). Our finding that expression of components of NADPH oxidase are increased in old IL-10-deficient mice is further evidence that oxidative stress is a key contributor to vascular abnormalities with aging. We assume that the changes in mRNA seen would be reflected in changes in levels of protein and oxidase activity. A novel aspect of this study is the finding that endogenous IL-10 may normally protect against increased expression of NADPH oxidase during aging. One of the limitations of this study is that we do not have data on local or circulating levels of IL-10. There is not much literature on effects of aging on IL-10 expression, but levels in tissue and plasma have been reported to decrease or not change with aging (Forsey et al. 2003; Saito et al. 2003; Frank et al. 2006).

Diverse mechanisms likely contribute to vascular abnormalities with aging. Although this study implicates an important role for oxidative stress, the findings do not rule out potential contributions by other mechanisms, particularly mechanisms that are driven by, or interact with, reactive oxygen species. Interactions between oxidant- and immune-related mechanisms are well described. Angiotensin II activates NF-*κ*B and increases expression of IL-6 (Schrader et al. 2007; Brasier 2010; Rojas et al. 2010; Johnson et al. 2013), whereas IL-6 promotes oxidative stress via activation of receptors for angiotensin II and NADPH oxidase (Wassmann et al. 2004; Dugan et al. 2009). Endothelial dysfunction in response to angiotensin II requires expression of both IL-6 and the Nox2 component of NADPH oxidase (Schrader et al. 2007; Chrissobolis et al. 2012). While this manuscript was in preparation, a report appeared suggesting that endothelial dysfunction occurs in older IL-10-deficient mice (but not wild-type) via a mechanism that involves cyclooxygenase and vasoconstrictor prostanoids (Sikka et al. 2013). That study focused on aorta and used mice at 9 months of age and older (a precise age was not provided) so detailed comparisons are difficult. However, interactions between oxidative stress and cyclooxygenase activity are known to exist (Faraci 2011b), so the findings in this study do not rule out potential contributions by other endothelium-dependent mechanisms in older IL-10-deficient mice. Overall, both studies support the concept that IL-10 exerts protective effects that suppress vascular aging.

In summary, oxidative stress appears to be a key component of mechanisms that underlie endothelial dysfunction with aging. This study provides evidence for accelerated oxidative stress and vascular aging in a mouse model that is genetically deficient in IL-10. Endothelial dysfunction in carotid arteries in this model was mediated by superoxide. Data from vascular tissue suggest that changes in expression of NADPH oxidase and IL-6 might contribute to the observed endothelial dysfunction in old IL-10-deficient mice. Collectively, these findings suggest that the balance between expression of IL-10 and mechanisms that promote vascular aging may be a key determinant of the progression of vascular disease. Abnormalities in endothelial cells contribute fundamentally to the onset and worsening of vascular disease. Approaches that target IL-10 or its downstream effectors may have beneficial therapeutic effects to suppress the progression of vascular diseases due to aging.

## References

[b1] Angulo J, Vallejo S, Garcia-Septiem M, El Assar J, Sanchez-Ferrer CF, Rodriguez-Manas L (2012). Age-related differences in the effects of *α* and *γ* peroxisome proliferator-activated receptor subtype agonists on endothelial vasodilation in human microvessels. Exp. Gerontol.

[b2] Arntzen KA, Mathiesen EB (2011). Subclinical carotid atherosclerosis and cognitive function. Acta Neurol. Scand. Suppl.

[b3] Boekholdt SM, Stroes ES (2012). The interleukin-6 pathway and atherosclerosis. Lancet.

[b4] Brasier AR (2010). The nuclear factor-kappaB-interleukin-6 signalling pathway mediating vascular inflammation. Cardiovasc. Res.

[b5] Brown KA, Chu Y, Lund DD, Heistad DD, Faraci FM (2006). Gene transfer of extracellular superoxide dismutase protects against vascular dysfunction with aging. Am. J. Physiol. Heart Circ. Physiol.

[b6] Brown KA, Didion SP, Andresen JJ, Faraci FM (2007). Effect of aging, MnSOD deficiency, and genetic background on endothelial function: evidence for MnSOD haploinsufficiency. Arterioscler. Thromb. Vasc. Biol.

[b7] Chrissobolis S, Banfi B, Sobey CG, Faraci FM (2012). Role of Nox isoforms in angiotensin II-induced oxidative stress and endothelial dysfunction in brain. J. Appl. Physiol.

[b8] Chu Y, Heistad DD, Knudtson KL, Lamping KG, Faraci FM (2002). Quantification of mRNA for endothelial NO synthase in mouse blood vessels by real-time polymerase chain reaction. Arterioscler. Thromb. Vasc. Biol.

[b9] Didion SP, Kinzenbaw DA, Schrader LI, Faraci FM (2006). Heterozygous CuZn superoxide dismutase deficiency produces a vascular phenotype with aging. Hypertension.

[b10] Didion SP, Kinzenbaw DA, Schrader LI, Chu Y, Faraci FM (2009). Endogenous interleukin-10 inhibits angiotensin II-induced vascular dysfunction. Hypertension.

[b11] Donato AJ, Eskurza I, Silver AE, Levy AS, Pierce GL, Gates PE (2007). Direct evidence of endothelial oxidative stress with aging in humans: relation to impaired endothelium-dependent dilation and upregulation of nuclear factor-kappaB. Circ. Res.

[b12] Drummond GR, Selemidis S, Griendling KK, Sobey CG (2011). Combating oxidative stress in vascular disease: NADPH oxidases as therapeutic targets. Nat. Rev. Drug Discov.

[b13] Dugan LL, Ali SS, Shekhtman G, Roberts AJ, Lucero J, Quick KL (2009). IL-6 mediated degeneration of forebrain GABAergic interneurons and cognitive impairment in aged mice through activation of neuronal NADPH oxidase. PLoS ONE.

[b14] El Assar M, Angulo J, Vallejo S, Peiro C, Sanchez-Ferrer CF, Rodriguez-Manas L (2012). Mechanisms involved in the aging-induced vascular dysfunction. Front Physiol.

[b15] Faraci F, Masoro EJ, Austad SN (2011a). Cerebral vascular dysfunction with aging. Handbook of the Biology of Aging.

[b16] Faraci FM (2011b). Protecting against vascular disease in brain. Am. J. Physiol. Heart Circ. Physiol.

[b17] Faraci FM, Sigmund CD, Shesely EG, Maeda N, Heistad DD (1998). Responses of carotid artery in mice deficient in expression of the gene for endothelial NO synthase. Am. J. Physiol.

[b18] Fleenor BS, Seals DR, Zigler ML, Sindler AL (2012). Superoxide-lowering therapy with TEMPOL reverses arterial dysfunction with aging in mice. Aging Cell.

[b19] Fogel B, Pober JS, Wick G, Grundtman C (2012). Vascular endothelial cells as immunological targets in atherosclerosis. Inflammation and Atherosclerosis.

[b20] Forsey RJ, Thompson JM, Ernerudh J, Hurst TL, Strindhall J, Johansson B (2003). Plasma cytokine profiles in elderly humans. Mech. Ageing Dev.

[b21] Frank MG, Barrientos RM, Biedenkapp JC, Rudy JW, Watkins LR, Maier SF (2006). mRNA up-regulation of MHC II and pivotal pro-inflammatory genes in normal brain aging. Neurobiol. Aging.

[b22] Gunnett CA, Berg DJ, Faraci FM (1999). Vascular effects of lipopolysaccharide are enhanced in interleukin-10-deficient mice. Stroke.

[b23] Gunnett CA, Heistad DD, Berg DJ, Faraci FM (2000). IL-10 deficiency increases superoxide and endothelial dysfunction during inflammation. Am. J. Physiol. Heart Circ. Physiol.

[b24] Gunnett CA, Heistad DD, Faraci FM (2002). Interleukin-10 protects nitric oxide-dependent relaxation during diabetes: role of superoxide. Diabetes.

[b25] Johnson AW, Kinzenbaw DA, Modrick ML, Faraci FM (2013). Small-molecule inhibitors of signal transducer and activator of transcription 3 protect against angiotensin II-induced vascular dysfunction and hypertension. Hypertension.

[b26] Lakatta EG, Levy D (2003). Arterial and cardiac aging: major shareholders in cardiovascular disease enterprises: aging arteries: a “set up” for vascular disease. Circulation.

[b27] Lamping KG, Faraci FM (2001). Role of sex differences and effects of endothelial NO synthase deficiency in responses of carotid arteries to serotonin. Arterioscler. Thromb. Vasc. Biol.

[b28] Lauer T, Preik M, Rassaf T, Strauer BE, Deussen A, Feelisch M (2001). Plasma nitrite rather than nitrate reflects regional endothelial nitric oxide synthase activity but lacks intrinsic vasodilator action. Proc. Natl. Acad. Sci. USA.

[b29] Libby P, Ridker PM, Hansson GK (2011). Progress and challenges in translating the biology of atherosclerosis. Nature.

[b30] Lorenz MW, Markus HS, Bots ML, Rosvall M, Sitzer M (2007). Prediction of clinical cardiovascular events with carotid intima-media thickness: a systematic review and meta-analysis. Circulation.

[b31] Lund DD, Chu Y, Miller JD, Heistad DD (2009). Protective effect of extracellular superoxide dismutase on endothelial function during aging. Am. J. Physiol. Heart Circ. Physiol.

[b32] Mayhan WG, Arrick DM, Sharpe GM, Sun H (2008). Age-related alterations in reactivity of cerebral arterioles: role of oxidative stress. Microcirculation.

[b33] Miwa K, Tanaka M, Okazaki S, Furukado S, Sakaguchi M, Mochizuki H (2013). Association between interleukin-6 levels and first-ever cerebrovascular events in patients with vascular risk factors. Arterioscler. Thromb. Vasc. Biol.

[b34] Modrick ML, Didion SP, Sigmund CD, Faraci FM (2009). Role of oxidative stress and AT1 receptors in cerebral vascular dysfunction with aging. Am. J. Physiol. Heart Circ. Physiol.

[b35] Modrick ML, Kinzenbaw DA, Chu Y, Sigmund CD, Faraci FM (2012). Peroxisome proliferator-activated receptor-gamma protects against vascular aging. Am. J. Physiol. Regul. Integr. Comp. Physiol.

[b36] Ouyang W, Rutz S, Crellin NK, Valdez PA, Hymowitz SG (2011). Regulation and functions of the IL-10 family of cytokines in inflammation and disease. Annu. Rev. Immunol.

[b37] Park L, Anrather J, Girouard H, Zhou P, Iadecola C (2007). Nox2-derived reactive oxygen species mediate neurovascular dysregulation in the aging mouse brain. J. Cereb. Blood Flow Metab.

[b38] Pena Silva RA, Chu Y, Miller JD, Mitchell IJ, Penninger JM, Faraci FM (2012). Impact of ACE2 deficiency and oxidative stress on cerebrovascular function with aging. Stroke.

[b39] Rodriguez-Manas L, El-Assar M, Vallejo S, Lopez-Doriga P, Solis J, Petidier R (2009). Endothelial dysfunction in aged humans is related with oxidative stress and vascular inflammation. Aging Cell.

[b40] Rojas M, Zhang W, Lee DL, Romero MJ, Nguyen DT, Al-Shabrawey M (2010). Role of IL-6 in angiotensin II-induced retinal vascular inflammation. Invest. Ophthalmol. Vis. Sci.

[b41] Rothwell PM, Coull AJ, Silver LE, Fairhead JF, Giles MF, Lovelock CE (2005). Population-based study of event-rate, incidence, case fatality, and mortality for all acute vascular events in all arterial territories (Oxford Vascular Study). Lancet.

[b42] Saito H, Sherwood ER, Varma TK, Evers BM (2003). Effects of aging on mortality, hypothermia, and cytokine induction in mice with endotoxemia or sepsis. Mech. Ageing Dev.

[b43] Schrader LI, Kinzenbaw DA, Johnson AW, Faraci FM, Didion SP (2007). IL-6 deficiency protects against angiotensin II induced endothelial dysfunction and hypertrophy. Arterioscler. Thromb. Vasc. Biol.

[b44] Sikka G, Miller KL, Steppan J, Pandey D, Jung SM, Fraser CD (2013). Interleukin 10 knockout frail mice develop cardiac and vascular dysfunction with increased age. Exp. Gerontol.

[b45] Tabas I, Glass CK (2013). Anti-inflammatory therapy in chronic disease: challenges and opportunities. Science.

[b46] Toth P, Tucsek Z, Sosnowska D, Gautam T, Mitschelen M, Tarantini S (2013). Age-related autoregulatory dysfunction and cerebromicrovascular injury in mice with angiotensin II-induced hypertension. J. Cereb. Blood Flow Metab.

[b47] Walker AE, Henson GD, Reihl KD, Nielson EI, Morgan RG, Lesniewski LA (2013). Beneficial effects of lifelong caloric restriction on endothelial function are greater in conduit arteries compared to cerebral resistance arteries. Age.

[b48] Wassmann S, Stumpf M, Strehlow K, Schmid A, Schieffer B, Bohm M (2004). Interleukin-6 induces oxidative stress and endothelial dysfunction by overexpression of the angiotensin II type 1 receptor. Circ. Res.

[b49] Wendell CR, Zonderman AB, Metter EJ, Najjar SS, Waldstein SR (2009). Carotid intimal medial thickness predicts cognitive decline among adults without clinical vascular disease. Stroke.

[b50] Wray DW, Nishiyama SK, Harris RA, Zhao J, McDaniel J, Fjeldstad AS (2012). Acute reversal of endothelial dysfunction in the elderly after antioxidant consumption. Hypertension.

